# Investigating the Role of Vanadium-Dependent Haloperoxidase Enzymology in Microbial Secondary Metabolism and Chemical Ecology

**DOI:** 10.1128/mSystems.00780-21

**Published:** 2021-08-24

**Authors:** Jackson T. Baumgartner, Shaun M. K. McKinnie

**Affiliations:** a Department of Chemistry and Biochemistry, University of California, Santa Cruz, Santa Cruz, California, USA

**Keywords:** biosynthesis, chemical ecology, enzymology, marine microbiology, natural antimicrobial products, vanadium-dependent haloperoxidase

## Abstract

The chemical diversity of natural products is established by an elegant network of biosynthetic machinery and controlled by a suite of intracellular and environmental cues. Advances in genomics, transcriptomics, and metabolomics have provided useful insight to understand how organisms respond to abiotic and biotic factors to adjust their chemical output; this has permitted researchers to begin asking bigger-picture questions regarding the ecological significance of these molecules to the producing organism and its community. Our lab is motivated by understanding how select microbes construct and manipulate bioactive molecules by utilizing vanadium-dependent haloperoxidase (VHPO) enzymology. This commentary will give perspective into our efforts to understand the unique VHPO-catalyzed conversions which modulate the activities within two ecologically relevant natural product families. Through enhancing our knowledge of microbial natural product biosynthesis, we can understand how and why these bioactive molecules are created.

## COMMENTARY

Natural products (NPs), also known as secondary or specialized metabolites, are composed of small organic molecules that have long been appreciated for their therapeutic potential (1). The marine ecosystem has been recognized as a rich source of novel NPs that can be leveraged against cancers, pathogenic bacteria, and other ailments. The genomes of microbes possess an incredible genetic potential to produce a plethora of secondary metabolites to defend themselves and interact with their environments (2, 3). The diversity found within NP scaffolds results from a number of biosynthetic gene clusters (BGCs) encoding unique NPs but also from singular BGCs producing multiple derivatives. There is good justification for why a single organism maintains several BGCs to produce divergent NP molecules (4); however, a unified evolutionary logic for why organisms maintain promiscuous secondary metabolic BGCs remains an open question (5, 6). Several excellent studies have provided insight into this question (7, 8) and have illuminated research into understanding not only how these derivatives are being produced but also why.

As the tools of chemical ecology have matured, researchers have been able to ask more targeted questions about the factors that elicit NP production. With modern genomic, transcriptomic, and metabolomic profiling techniques, the field has started to reify links between secondary metabolite production and the specific internal state of a single organism while considering its surrounding community. The signal-molecule-based activation of silent NP BGCs has emphasized the role that the local microbiota plays in the elicitation of diverse community-modulating NPs (9, 10). Our lab is passionate about investigating the precise mechanisms of how bioactive microbial NPs are generated and diversified at the species level. However, we are becoming increasingly curious about the biotic and abiotic factors that elicit NP production and the roles these specialized metabolites play within their local environments. To that effect, we are particularly interested in understanding the impact of vanadium-dependent enzymology in marine NP biosynthesis and chemical ecology.

**FIG 1:**
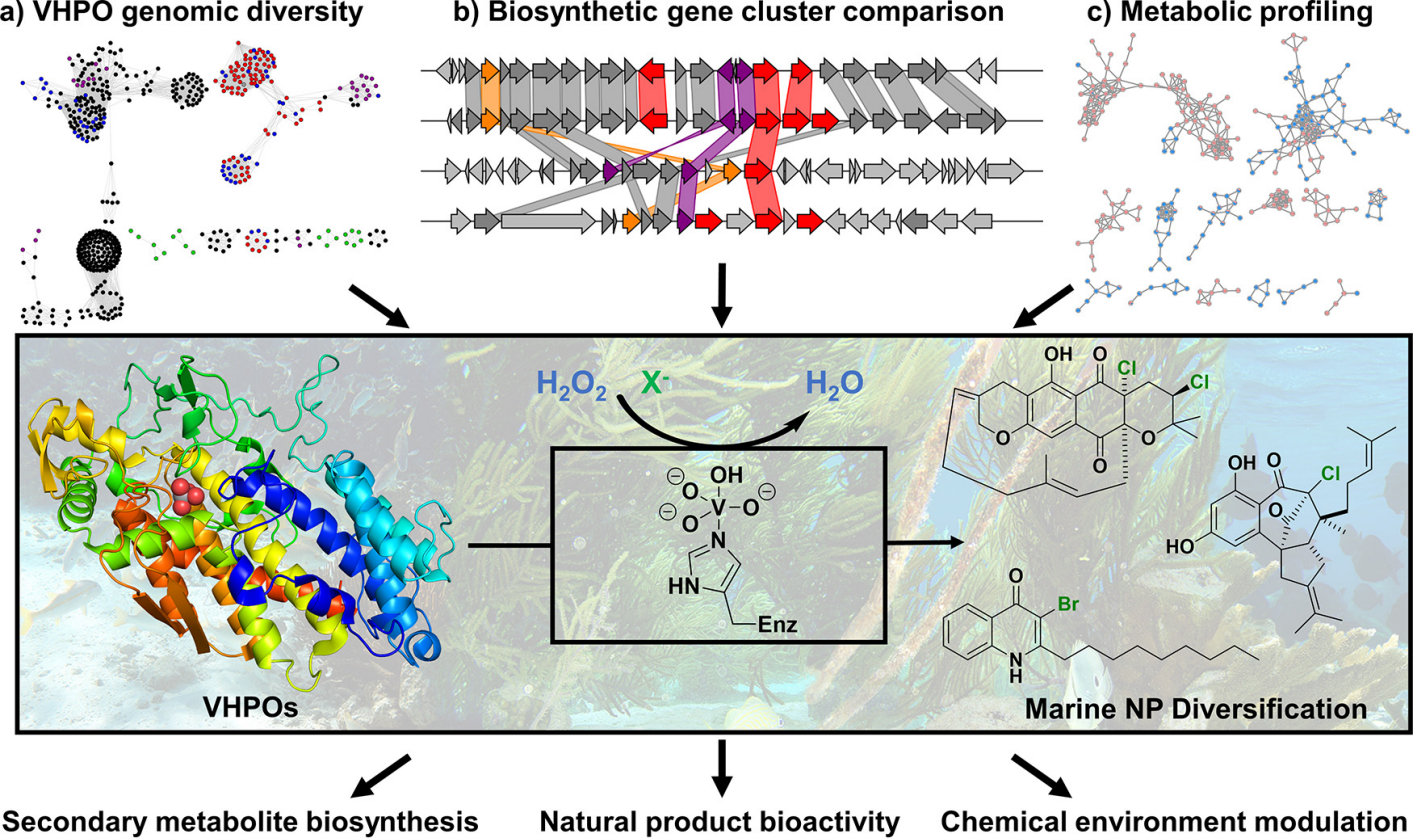
General research overview of our approach to understanding the role of vanadium-based enzymology in modifying microbial intracellular and community chemical environments. We aim to (a) utilize Enzyme Function Initiative tools to identify and categorize microbial VHPO homologs based on sequence similarities (20), (b) investigate the genomic context of VHPO genes to provide insight into putative enzyme function and NP substrate scaffolds (figure generated by Clinker [24]), and (c) apply tandem mass spectrometry (MS-MS)-based molecular networking techniques (25) to facilitate rapid interrogation of the metabolic scope of VHPO-containing microbes and their environments.

One of the many unique features of the marine biosphere is the relative abundance of vanadium and its subsequent utilization by microbial and macroalgal communities (11). In the form of vanadate, this trace metal plays several roles within marine organisms including nitrogen fixation, bacterial respiration, and accumulation in select eukaryotes (12). Our interests in this field involve a unique family of enzymes, the vanadium-dependent haloperoxidases (VHPOs), which use a vanadate prosthetic group and reactive oxygen species (primarily hydrogen peroxide) to oxidize halide ions to hypohalous acids. Macroalgal and cyanobacterial VHPOs have been the earliest and most extensively studied homologs (13) and generally produce freely diffusible hypohalous acid that reacts nonspecifically with organic molecules based on their inherent chemical reactivities. Nonselective VHPOs play a significant ecological role for their host organisms through general antifouling/antigrazing mechanisms, exogenous molecule degradation, and iodine gas production in response to oxidative stress (13); however, they are most notably implicated in the production of volatile haloforms (chloroform and bromoform), of which the environmental impacts remain to be fully understood (14). In contrast, VHPOs from *Streptomyces* and other bacteria site-specifically halogenate small molecules to elicit precise biosynthetic transformations (15). We intend to use a multi-omics approach to understand how these underexplored site-specific bacterial VHPO enzymes play a role in the chemical elaboration of NP scaffolds and also how this chemodiversity plays a role in establishing the ecological niche of the host microbe (Fig. 1).

**FIG 2:**
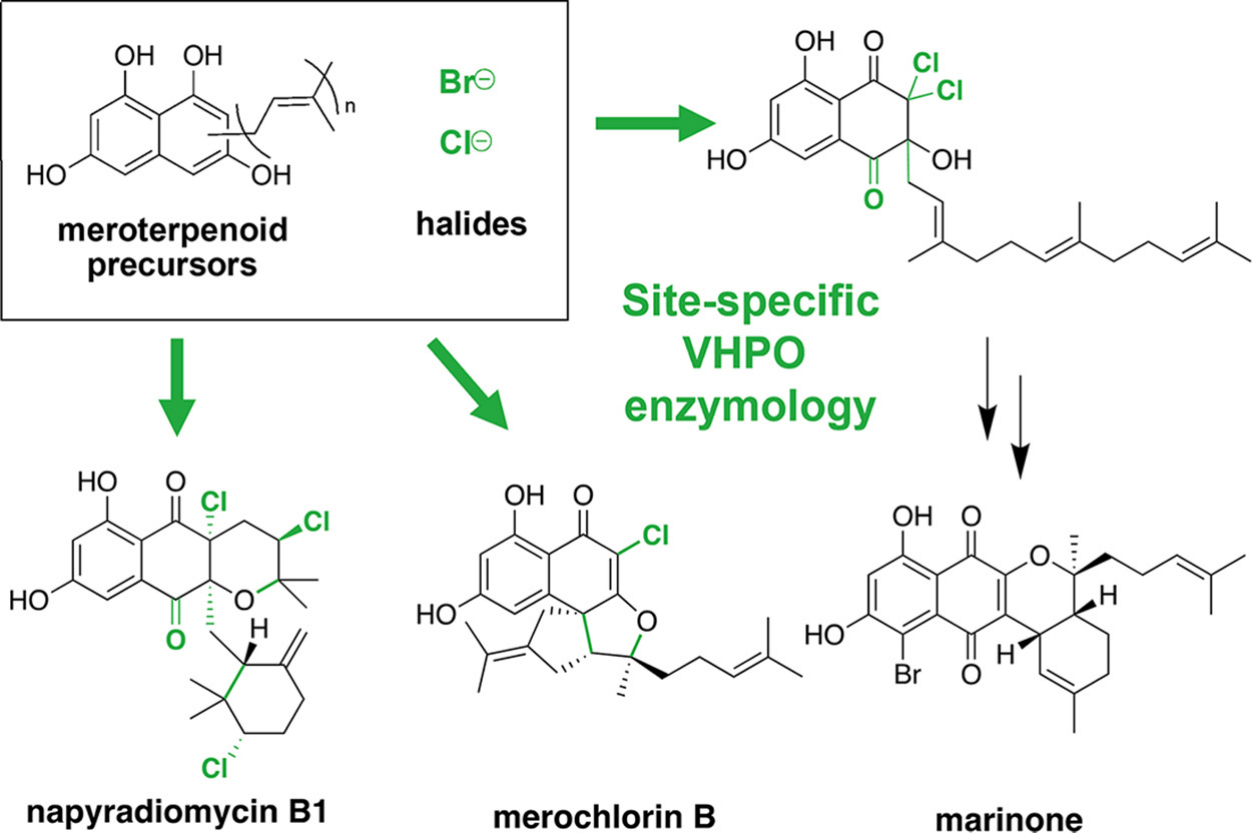
Site-specific VHPO enzymology plays a significant role in establishing the chemical complexity within actinobacterial meroterpenoid antibiotic NPs. Key VHPO-catalyzed transformations from the napyradiomycin (17), merochlorin (18), and marinone (19) biosynthetic pathways are highlighted in green.

The most explored area of site-specific VHPO biosynthetic enzymology has involved the diverse naphthoquinone-based family of meroterpenoids. These mixed polyketide-terpene NPs are produced by marine and soil actinobacteria and exhibit a range of useful Gram-positive antibacterial, cytotoxic, and antifouling activities (16). Despite their divergent chemical structures, these NPs are assembled from similar precursors: a 1,3,6,8-tetrahydroxynaphthalene (THN) core, variable-length isoprenoid appendages, and halogen atoms. Site-specific VHPOs play a significant role in derivatizing and diversifying the chemical structures and bioactivities within these meroterpenoid scaffolds, as exemplified within the biosyntheses of the napyradiomycins (17), merochlorins (18), and marinone (19) (Fig. 2).

Of the THN-derived meroterpenoids, the napyradiomycins are uniquely well suited for hypothesizing “how-and-why” NPs are interconverted by VHPOs and other accessory enzymes in the marine biosphere. Over 50 unique napyradiomycins have been isolated, and new molecules continue to be discovered (16). Some of these derivatives likely arise from isolation and purification processes, although many are intentionally generated by the bacteria to modulate the inherent bioactivity of the NP itself. The napyradiomycins can be subdivided into seven main structural classes that have variable antimicrobial activities which do not correlate with progression along the established NP biosynthetic pathway. Intermediate NPs like napyradiomycin A1 can be converted into the potent antimicrobial napyradiomycin B1 through the action of VHPO NapH4 (17) or can be macrocyclized to the less active napyradiomycin C/D/SR derivatives through an unknown mechanism. From a traditional NP perspective, these less potent molecules are little more than synthetically challenging oddities; however, we hypothesize that these derivatives assist the napyradiomycin producer to modulate its interactions with its community and establish its ecological niche. We are interested in the role of additional uncharacterized VHPO enzymology in meroterpenoid NP interconversions and also the impact of this derivatization on marine microbial communities. Preliminary analyses using sequence similarity and genomic neighborhood analyses (20) have indicated that site-specific VHPO enzymes are present in a variety of other bacterial genera with differing genomic contexts (Fig. 1); we aim to fully explore the impact of VHPO enzymology not only in *Streptomyces* NP biosynthesis but also within less established marine and terrestrial microbes.

Although less established than their roles in constructing endogenous NPs via biosynthetic routes, there is increasing evidence to suggest that VHPOs may modulate exogenous quorum sensing molecules. A macroalgal VHPO has been previously established to disrupt bacterial communication through homoserine lactone degradation and bromofuranone production to minimize surface fouling (21). The alkyl quinolones (AQs) are an abundant group of quorum sensors known to contribute to the pathogenesis of Pseudomonas species (22). These metabolites have been investigated for their antimicrobial activities and show an intriguing synergistic effect when multiple AQ derivatives are used in combination (8). We are particularly interested in the recent discovery of a marine gammaproteobacterial VHPO capable of site-specifically brominating select AQs; this simultaneously detoxified these metabolites toward the *Microbulbifer* host species while increasing their toxicity toward other bacteria (23). This VHPO and its homologs in other Gram-negative proteobacteria are not localized to biosynthetic gene clusters, and outside the originally characterized microbe, no other source organisms appear to possess the genetic potential to endogenously produce AQs. This subclass of marine bacterial VHPOs appears to be directly involved in the modification of microbial community environmental cues. This hypothesis is further supported by the conserved presence of secretion signal sequences appended to these VHPO genes, implying the extracytosolic localization of these enzymes.

We intend to initially investigate the impact of AQ-modulating VHPOs using *in vitro* enzymology with ecologically relevant marine bacterial homologs. Pending the generalizability of this halogenation biochemistry, we aim to begin assessing the *in vivo* activities of these VHPOs in axenic cultures and within simulated communities alongside known AQ producers. Transcriptomic and metabolomic analyses using liquid coculturing experiments may provide insight into the role of VHPO-mediated AQ modification. Imaging mass spectrometry in combination with solid-phase coculturing conditions can additionally assess the spatial localization of quorum sensor production, interconversion, and microbial interactions. We envision that a rigorous understanding of AQ halogenation enzymology will enable us to target specific ecological questions regarding the significance of VHPOs in marine microbiology.

In comparison to common terrestrial transition metals like iron, molybdenum, and zinc, the full extent of the biological and ecological roles that vanadium plays is less well understood (11, 12). Much of the biochemistry underpinning vanadate acquisition and utilization remains underexplored for such an accessible marine metal. In the past decade since their biochemical validation, site-specific actinobacterial VHPO homologs have been shown to catalyze a diverse array of chemical reactions (15–19), including chiral halogenations, forming new carbon-oxygen and carbon-carbon bonds, aromatic dearomatization reactions, and, perhaps most intriguingly, NP skeletal rearrangements. A major motivation of our future work involves leveraging these unique transformations biocatalytically to efficiently and cleanly manufacture bioactive NPs and their analogs. From a systems microbiology viewpoint, focusing on the biochemistry enabled by the presence of vanadium provides a unique perspective on the drivers of marine ecology and the maintenance of microbial niches. We propose that investigating the chemistry enabled by VHPOs will not only provide unique insight into NP discovery and biosynthesis but also uncover new roles for previously discovered scaffolds and new mechanisms of environmental modulation. Advances in genomics, metabolomics, and analytical chemistry have permitted us to better visualize the unexplored genetic and chemical space occupied by bacterial VHPOs, illuminating many lucrative routes to interrogate the impact of vanadium in the biosphere.
